# An Exploratory Endoscopic Achalasia Phenotype With Visible Squamocolumnar Junction and Distal Esophagogastric Junction Relaxation Failure: Association With Gastroesophageal Reflux Disease After Peroral Endoscopic Myotomy (With Video)

**DOI:** 10.1111/den.70149

**Published:** 2026-04-04

**Authors:** Kazuya Sumi, Haruhiro Inoue, Yohei Nishikawa, Kazuki Yamamoto, Ippei Tanaka, Mayo Tanabe, Manabu Onimaru, Koji Otsuka, Takayoshi Ito, Noboru Yokoyama

**Affiliations:** ^1^ Digestive Diseases Center Showa Medical University Koto‐Toyosu Hospital Tokyo Japan

**Keywords:** achalasia, gastroesophageal reflux disease, peroral endoscopic myotomy, squamocolumnar junction

## Abstract

**Backgrounds and Aims:**

Esophageal achalasia is typically defined by impaired lower esophageal sphincter (LES) relaxation. In rare cases, complete circumferential visibility of the squamocolumnar junction (SCJ) and upper gastric folds (GFs) is seen endoscopically, suggesting functional obstruction may extend to the distal esophagogastric junction rather than being confined to the LES. Such cases appear to be associated with a high risk of gastroesophageal reflux disease (GERD) after peroral endoscopic myotomy (POEM). We clarified the characteristics and significance of this rare endoscopic phenotype.

**Methods:**

We retrospectively reviewed the data of 2553 patients who underwent POEM at our center between 2014 and 2023, and selected cases with full SCJ visibility and relaxation failure at the upper border of the GF before POEM. Patients with prior endoscopic or surgical treatments were excluded. Demographic, procedural, and GERD data were analyzed. GERD severity was assessed endoscopically.

**Results:**

Twelve patients (0.7%) met these criteria. All were male, with a median age and body mass index of 48.5 years and 21.7 kg/m^2^, respectively. None had a hiatal hernia. All had straight‐type achalasia without advanced esophageal dilation. Median myotomy lengths were 7.0 (esophageal) and 4.0 (gastric) cm, respectively. Endoscopic esophagitis was observed in 92% and conclusive GERD in 75%, markedly exceeding previous rates. There was no significant difference in GERD incidence between the anterior and posterior myotomy approaches.

**Conclusions:**

These endoscopic findings may represent achalasia with distal relaxation failure and a high risk of conclusive GERD after POEM. Identifying this endoscopic feature is important for procedural planning.

**Abbreviations**
BD: Balloon dilation
BMI: Body mass index
CGS: Champagne Glass Sign
DES: Diffuse esophageal spasm
EGD: Esophagogastroduodenoscopy
EGJ: Esophagogastric junction
GERD: Gastroesophageal reflux disease
GF: Gastric fold
HH: Hiatal hernia
HRM: High‐resolution manometry
IQRs: Interquartile ranges
IRP: Integrated relaxation pressure
LES: Lower esophageal sphincter
PCAB: Potassium‐competitive acid blockers
POEM: Peroral endoscopic myotomy
PPI: Proton‐pump inhibitors
RS: Rosette sign
SCJ: Squamocolumnar junction

## 1 Introduction

Esophageal achalasia, a primary motility disorder characterized by impaired lower esophageal sphincter (LES) relaxation, has traditionally been treated with balloon dilation (BD) and Heller–Dor surgery. Since its introduction, peroral endoscopic myotomy (POEM) has demonstrated wide efficacy and is now the standard treatment for achalasia [1]. POEM has also been successfully applied to other esophageal motility disorders [2], showing comparable therapeutic outcomes to surgical treatment [3].

Although POEM is a minimally invasive procedure, gastroesophageal reflux disease (GERD) remains a significant concern after treatment. However, multicenter studies indicated that most post‐POEM GERD cases are mild and manageable with acid‐suppressive medications. Nonetheless, severe GERD is relatively uncommon, occurring in approximately 5%–12% of patients post‐POEM, with previous treatment and longer myotomy length being its risk factors [4, 5, 6].

Our institution, the birthplace of POEM, has performed approximately 3000 procedures. Through this extensive experience, we have identified rare but distinctive achalasia cases where the entire squamocolumnar junction (SCJ) and the upper border of the gastric folds (GFs) are clearly visible in the forward endoscopic view. This presentation lacks typical achalasia endoscopic features, including the rosette sign (RS) (seen in the forward view [7]) and champagne glass sign (CGS) (seen in retroflexion [8]).

For instance, in one of our cases, impaired relaxation was noted not at the conventional LES level but at the upper border of the GFs [9, 10]. Following POEM, the patient developed conclusive GERD, which resolved with conservative management, indicating a subset of achalasia where relaxation failure extended further toward the distal end of the esophagogastric junction (EGJ).

Recently, the EGJ has been reconceptualized as a three‐phase functional antireflux barrier: Phase I, mainly formed by the collar sling and clasp fibers constituting the gastroesophageal flap valve; Phase II, corresponding to the LES; and Phase III, related to esophageal clearance [11]. This “phase concept” explains the EGJ pathology beyond traditional LES dysfunction. In this context, we hypothesized that achalasia cases with a visible SCJ and distal relaxation failure might represent a distinct anatomical subtype characterized by predominant Phase I involvement.

In the present study, we aimed to identify and analyze cases with this unusual endoscopic phenotype, characterized by visible SCJ and relaxation failure at the distal end of the EGJ, to better understand its clinical features and implications for POEM planning and postoperative GERD risk.

## 2 Methods

### 2.1 Study Design

This retrospective single‐center cohort study enrolled 2553 patients who underwent POEM at Showa University Koto‐Toyosu Hospital between April 2014 and March 2023. The present study was approved by the Institutional Review Board of Showa University Koto‐Toyosu Hospital (approval number: 2024‐130‐B) and was conducted in accordance with the principles stipulated in the Declaration of Helsinki.

The POEM indication was determined based on a comprehensive evaluation using high‐resolution manometry (HRM; Starlet system, Star Medical, Tokyo, Japan), esophagram, and esophagogastroduodenoscopy (EGD) under sedation. All preoperative endoscopic examinations were performed under standardized propofol‐based sedation according to the institutional protocol. The depth of sedation was maintained at a level corresponding to moderate to deep sedation (Modified Observer's Assessment of Alertness/Sedation score 2–3) in all cases.

We first reviewed all patients who underwent POEM during the study period. To minimize selection bias, all patients who underwent POEM during the study period were reviewed and screened without exclusions beyond the predefined criteria. From this population, patients who met the following inclusion criteria were selected as the main study cohort:

1. a diagnosis of esophageal achalasia confirmed by esophageal stasis on esophagram or elevated integrated relaxation pressure (IRP) on HRM;
2. no prior history of POEM, BD, or Heller–Dor surgery; and
3. the full circumference of the SCJ clearly visible in the forward view before the endoscope passed through the EGJ and relaxation failure was observed at the upper border of the GF.

Relaxation failure at the upper border of the GFs was defined as persistent resistance to forward passage of the endoscope at the fold line despite gentle insufflation, with full circumferential visibility of the SCJ before EGJ traversal. Pre‐POEM endoscopy was independently reviewed by two experienced endoscopists according to this predefined operational definition; discrepancies were adjudicated by a third reviewer.

We excluded patients with typical endoscopic signs of achalasia (e.g., RS or CGS), those who underwent POEM for other motility disorders (e.g., diffuse esophageal spasm, Jackhammer esophagus), those with contraindications to general anesthesia (e.g., severe ischemic heart disease, chronic kidney disease, liver cirrhosis, or significant respiratory disease), pregnant or lactating women, those with morbid obesity, those with major psychiatric disorders, or those with a history of substance abuse.

### 2.2 POEM Procedure

POEM was performed under general anesthesia. All procedures were performed by experienced endoscopists, independently or under direct supervision of senior faculty. After a submucosal injection of a saline solution mixed with indigo carmine, a mucosal incision was made, followed by submucosal tunnel creation. The circular muscle layer was dissected under direct visualization, and the longitudinal muscle was preserved as much as possible. During the POEM procedure, a double‐scope method was used to confirm both the distal extent and the orientation of the submucosal tunnel and myotomy. The gastric myotomy endpoint was set 1–2 cm distal to the narrowest EGJ segment under double‐scope guidance, and the gastric myotomy length was measured from the SCJ in forward view. This technique also allowed confirmation that the tunnel direction did not deviate toward the greater curvature, thereby avoiding unintended injury to the gastric sling fibers. After completion of the procedure, the mucosal incision was closed with clips. All patients received proton‐pump inhibitors (PPIs) for 1 month after POEM, which were then completely discontinued. At 2 months after POEM, a follow‐up evaluation was conducted using EGD, HRM, and esophagram.

### 2.3 Outcome Measures

The details of the following parameters were collected: age, sex, body mass index (BMI), duration of symptoms, subjective symptoms, HRM classification, IRP, presence of hiatal hernia (HH), esophageal dilation (diameter and grade), and achalasia type (straight or sigmoid). The esophageal dilatation grade was classified as follows: grade I (< 3.5 cm), grade II (3.5–6.0 cm), or grade III (> 6.0 cm), based on the esophagram findings using the Japan Society of Esophageal Diseases criteria [12]. The EGD, HRM, and esophagram data as well as subjective symptom scores were obtained for all patients before and after POEM. Symptom severity was evaluated using the Eckardt score [13]. The HRM diagnosis was based on the Chicago Classification criteria v 3.0 [14]. For the POEM, we evaluated the myotomy length (total, esophageal side, and gastric side), incision axis, and utilization of the double‐scope method.

GERD severity was assessed at 2 months after POEM using the Los Angeles (LA) classification [15]. Conclusive GERD was defined as LA grade C or D esophagitis according to the Lyon Consensus [16]. Additionally, sustained use of acid suppressants [PPIs or potassium‐competitive acid blockers (PCABs)] over the 2 months was determined.

### 2.4 Statistical Analysis

Categorical variables were presented as frequencies and percentages, whereas continuous variables were expressed as medians with interquartile ranges (IQRs), owing to non‐normal distributions. The Wilcoxon signed‐rank test was used to compare the paired continuous variables (e.g., pre‐ and post‐treatment Eckardt scores). The Fisher's exact test was used to examine the associations between categorical variables, such as the incision axis and incidence of conclusive GERD. *p* values of < 0.05 were considered statistically significant. All statistical analyses were performed using JMP Pro version 16 (SAS Institute Inc., Cary, NC, USA). In addition, an exploratory comparison was performed between patients with visible SCJ and the remaining achalasia cohort with available postoperative endoscopic data, focusing on myotomy length and the incidence of erosive esophagitis. Interobserver agreement was assessed using Cohen's kappa statistic.

## 3 Results

**FIGURE 1:**
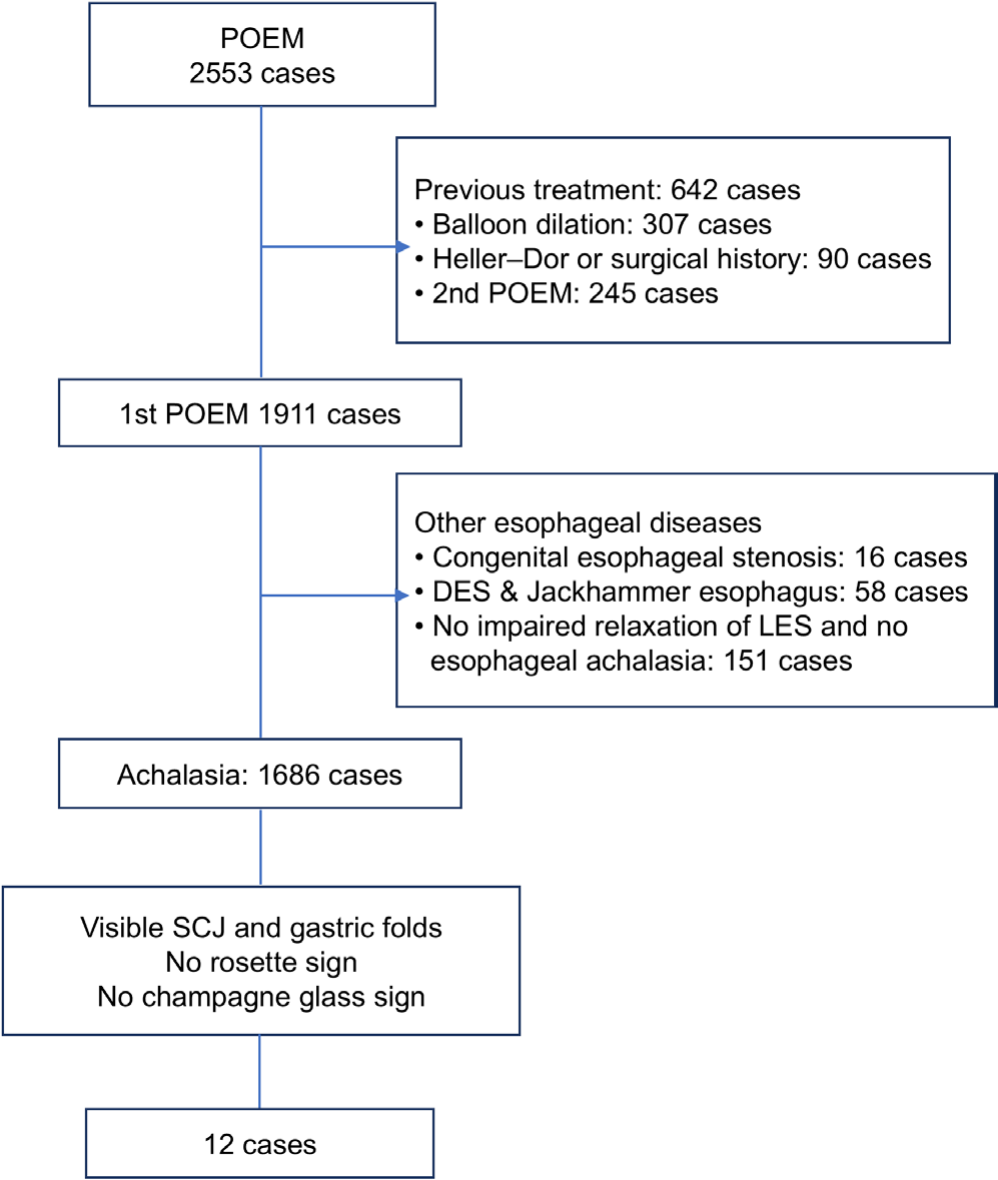
Flow diagram of the selection process. DES, Diffuse esophageal spasm; LES, Lower esophageal sphincter; POEM, Peroral endoscopic myotomy; SCJ, Squamous columnar junction.

During the study period, 2553 patients underwent POEM; of these cases, 642 cases with prior treatment (BD, Heller–Dor surgery, or second POEM) and 225 cases with nonachalasia esophageal motility disorders were excluded. As a result, 1686 patients with untreated esophageal achalasia were identified as the main cohort. Endoscopic findings were reviewed in all of these cases, and 12 patients with full circumferential visibility of the SCJ and relaxation failure of the GFs were selected for detailed analysis (Representative endoscopic findings are shown in Video S1). These patients did not exhibit the typical RS or CGS on preoperative endoscopy (Figure 1). Two endoscopists independently screened all 1686 cases for the visible SCJ phenotype, showing high interobserver agreement (Cohen's kappa = 0.96). One discrepant case was adjudicated by a third reviewer. Of the 1686 patients in the main cohort, postoperative endoscopic follow‐up data were available in 1559 after exclusion of cases with missing values. These 1559 patients were used as the reference cohort for exploratory comparison with the visible SCJ group (*n* = 12).

Baseline demographic, clinical, and manometric characteristics are summarized in Table 1. All patients were male and had straight‐type achalasia with impaired EGJ relaxation and absent or abnormal peristalsis. None had advanced esophageal dilation or a hiatal hernia. Clinical symptoms, assessed by the Eckardt score, improved significantly after POEM.

**TABLE 1:** Characteristics of the study population (*n* = 12).

Baseline characteristics	
Age, years, median [IQR]	48.5 [41.0–58.8]
Sex (male), *n* (%)	12/12 (100%)
BMI, kg/m^2^	21.7 [20.7–23.6]
Symptom duration before treatment, months, median [IQR]	12.0 [5.5–48.0]
Esophagram (straight; sigmoid)	12; 0
Esophageal dilation grade (I; II; III)	3; 9; 0
Esophageal diameter, cm, median [IQR]	4.2 [3.6–4.8]
High‐resolution manometry (pre‐POEM)	
Chicago classification (I; II; III)	7; 4; 1
IRP, mmHg, median [IQR]	32.5 [24.5–41.7]
Hiatus hernia, *n* (%)	0/12 (0%)
Eckardt score (pre‐ and post‐POEM)	
Pre‐POEM	6.5 [4–7]*
Post‐POEM	0.5 [0–1.3]*

Abbreviations: BMI, body mass index; IRP, integrated relaxation pressure; POEM, peroral endoscopic myotomy.

* Pre‐POEM vs. Post‐POEM; *p*‐value < 0.001.

Procedural details, including myotomy length, incision axis, and use of the double‐scope technique, are summarized in Table 2. The median myotomy lengths were 7 cm [IQR: 5–8.3 cm] and 4 cm [IQR: 3–4] on the esophageal and gastric sides, respectively, resulting in a total myotomy length of 10.5 cm [IQR: 9–12] cm. For exploratory comparison, the gastric myotomy length was significantly longer in the visible SCJ group than in the reference cohort (4 cm [IQR 3–4] vs. 3 cm [IQR 3–3], *p* = 0.026), whereas the esophageal myotomy length did not differ significantly between the two groups (7 cm vs. 8 cm, *p* = 0.24).

**TABLE 2:** POEM characteristics (*n* = 12).

Myotomy length, cm (esophageal side), median [IQR]	7 [5–8.3]
Myotomy length, cm (gastric side), median [IQR]	4 [3–4]
Myotomy length, cm (total), median [IQR]	10.5 [9–12]
Double‐scope method, *n* (%)	12/12 (100%)
Incision axis (anterior; posterior)	5; 7
PPI or P‐CAB administration after POEM, *n* (%)	9/12 (75%)

Abbreviations: P‐CAB, potassium‐competitive acid blocker; POEM, peroral endoscopic myotomy; PPI, proton‐pump inhibitor.

**FIGURE 2:**
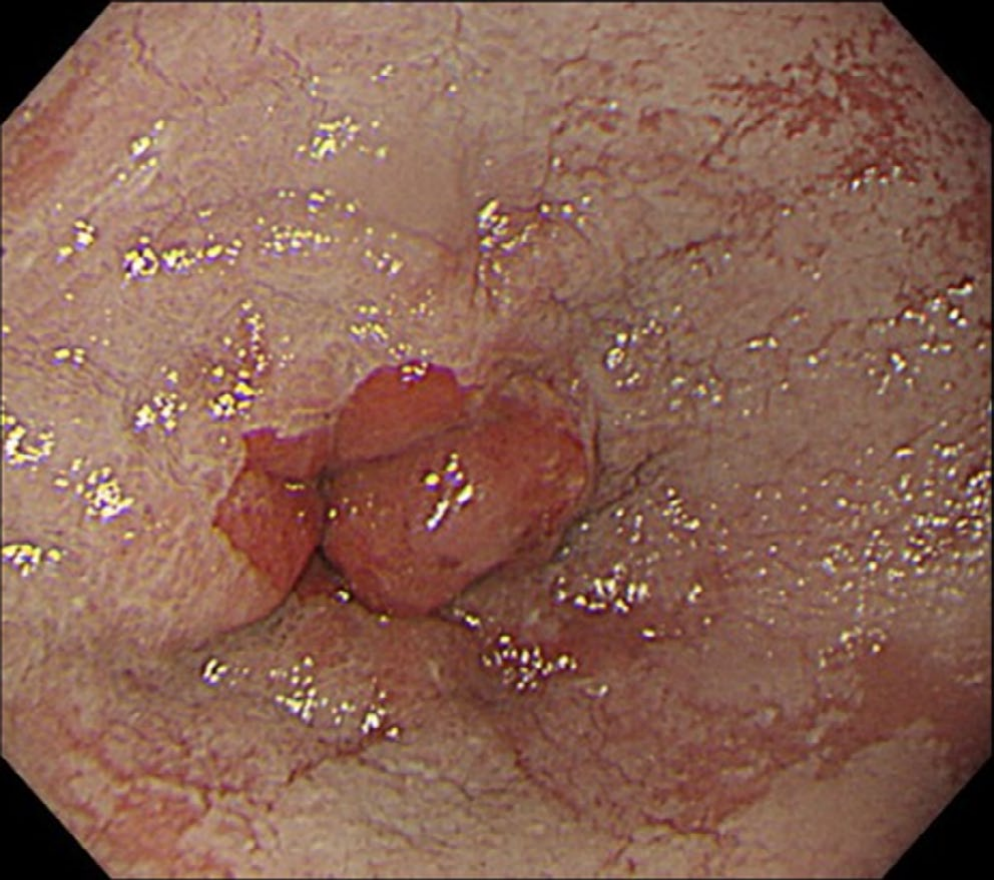
Endoscopic images before peroral endoscopic myotomy (POEM). A mid‐range endoscopic view clearly showing the squamocolumnar junction (SCJ), with impaired relaxation of the gastric folds (GFs).

**FIGURE 3:**
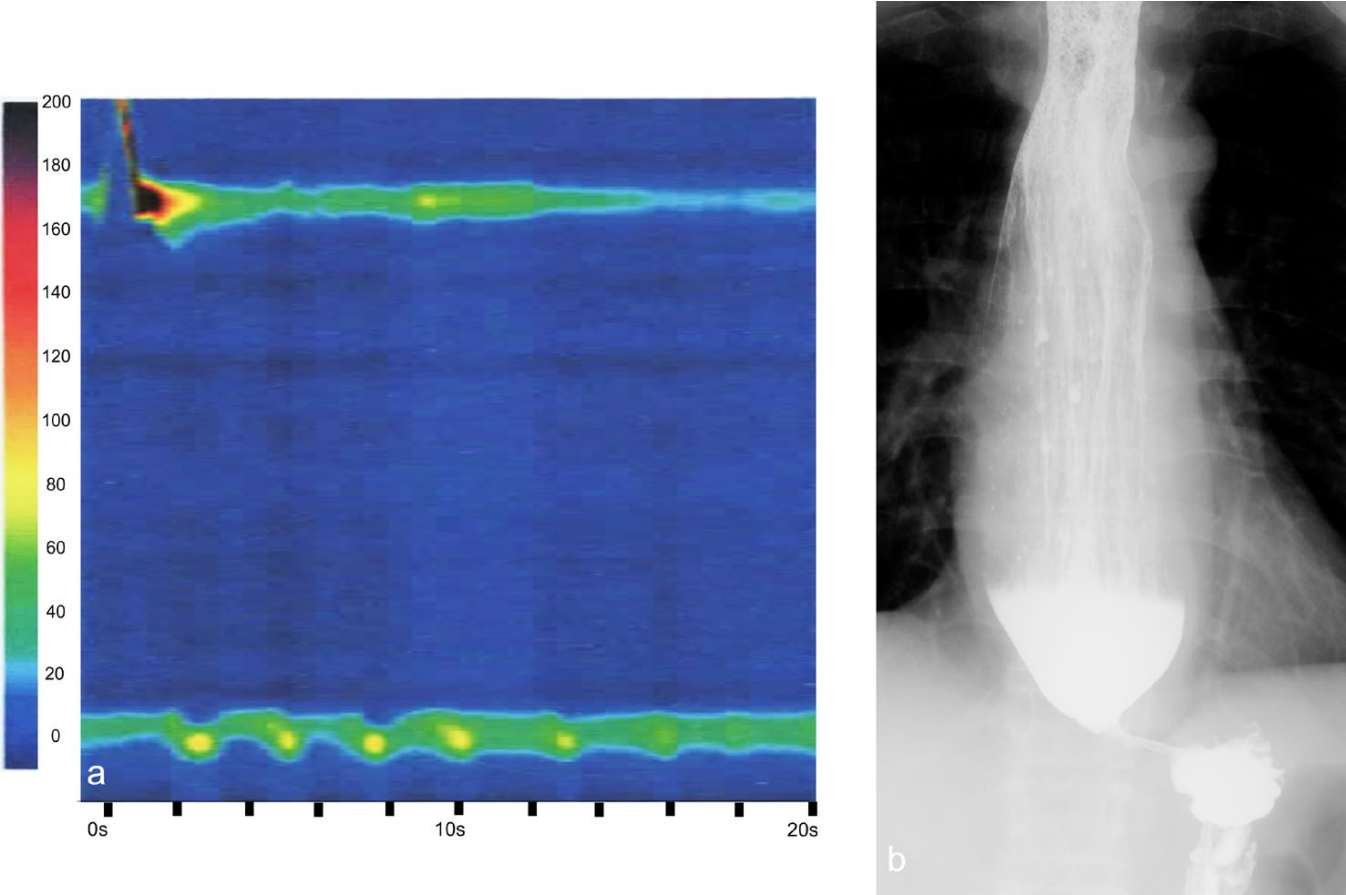
High‐resolution manometry (HRM) image and esophagram. (a) HRM image showing absent contractility and impaired relaxation of the lower esophageal sphincter (LES). (b) An esophagogram demonstrating barium stasis and impaired passage.

**FIGURE 4:**
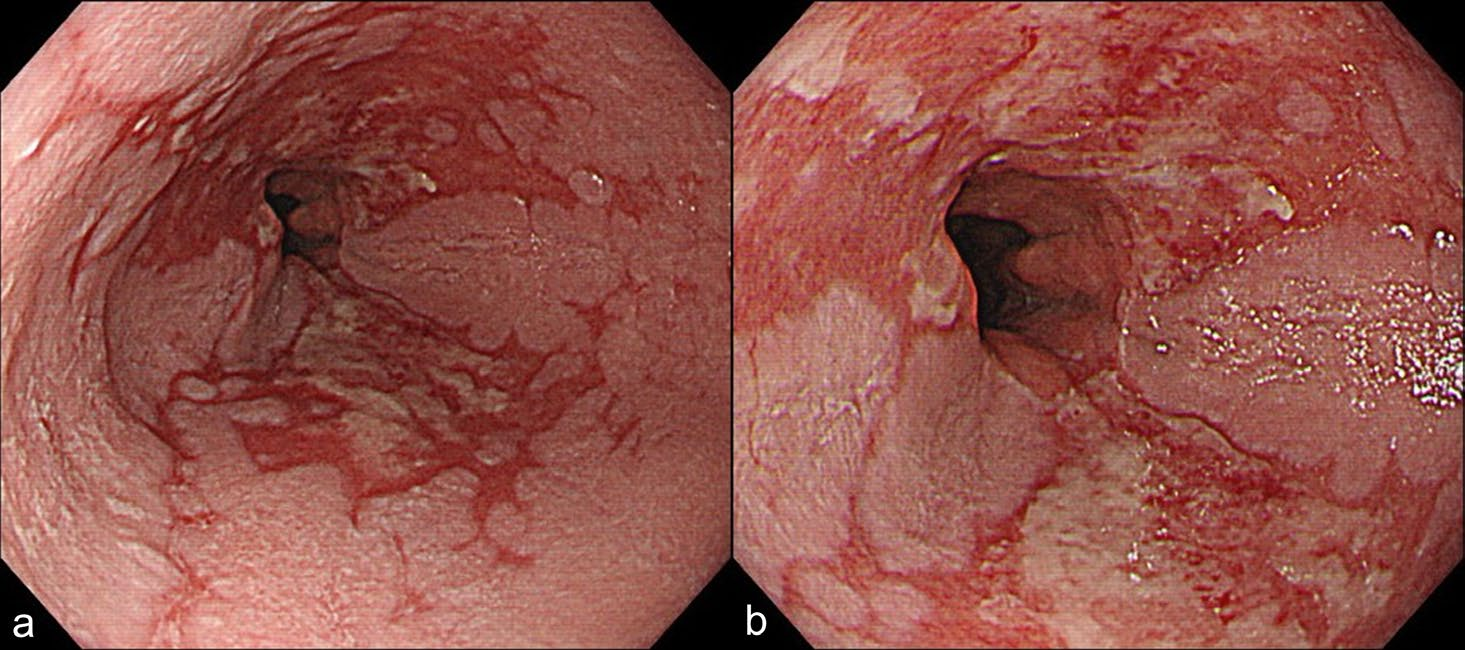
Endoscopic images after POEM demonstrating Los Angeles grade D esophagitis. (a) Mid‐range endoscopic view. (b) Close‐up view of the same lesion.

Two months after the POEM, erosive esophagitis was observed in 11 of 12 patients (92%). Among them, 9 of the 12 (75%) developed conclusive GERD, defined as LA grade C or D esophagitis, with 3 and 6 patients having grade C and D, respectively (Figures 2, 3, 4). The remaining two patients (17%) had mild erosive esophagitis, with one case each of LA grade A and B. However, the incidence of conclusive GERD was not significantly different between the anterior and posterior incision groups (80% vs. 71%, *p* = 1.00). In the reference cohort, conclusive GERD was observed in 45 of 1559 patients (2.9%), which was significantly lower than in the visible SCJ group (*p* < 0.0001). All patients with conclusive GERD continued PPI or P‐CAB therapy beyond 2 months. Most of them responded to conservative treatment; however, long‐term follow‐up revealed that the majority of patients continued to take either a PPI or P‐CAB.

## 4 Discussion

The present exploratory study revealed that esophageal achalasia cases with endoscopically visible circumferential SCJ and relaxation failure at the upper edge of the GFs are extremely rare, accounting for only 0.7% of our treated achalasia cohort. Among these patients, 92% (11/12) developed erosive esophagitis. The fact that 75% of these male patients developed conclusive GERD following POEM, especially given the lack of prior treatment, absence of sigmoid esophagus, nonelevated Eckardt scores, and absence of HH, raises the possibility that this endoscopic feature may be a visual predictor of a high risk for post‐POEM GERD. Importantly, the established risk factors for post‐POEM GERD, including ≥ 65 years, prior interventions, high Eckardt scores, presence of a sigmoid esophagus, long esophageal myotomy, and female sex, were absent in our cases, yet they still developed LA grade C/D esophagitis [4, 5, 6].

Based on previous studies, the incidence of endoscopic GERD after POEM ranges from approximately 20%–65%, with LA grade C/D esophagitis occurring in approximately 5%–12% of cases [4, 5, 6]. Notably, the exceptionally high rate of LA grade C/D esophagitis in our cohort underscores the potential relevance of this rare endoscopic phenotype. Although various risk factors, including gastric myotomy length and sling fiber injury, have been implicated in post‐POEM GERD, pre‐POEM endoscopic predictors have not been well characterized [17]. Our findings suggest that visible SCJ with relaxation failure on the gastric side may represent a previously unrecognized risk marker.

Post‐POEM GERD is known to be multifactorial. Reported risk factors include excessive extension of the gastric myotomy, disruption of the intrinsic antireflux barrier at the EGJ, impairment of the sling and clasp fibers, hypotensive LES after myotomy, HH, and alteration of the angle of His. In the present series, none of the patients had a HH, and the sling fibers were preserved under double‐scope guidance regardless of the anterior or posterior approach. Nevertheless, a remarkably high incidence of conclusive GERD was observed. This suggests that, in this specific phenotype, the functional obstruction and impaired relaxation may be located at the distal end of the EGJ high‐pressure zone and that extended distal myotomy required to relieve this obstruction may lead to substantial impairment of the intrinsic EGJ barrier, thereby predisposing patients to severe reflux even in the absence of classical anatomical risk factors.

No significant difference in GERD incidence was observed among the cases stratified based on the axis of myotomy. Notably, although the gastric endpoint of the myotomy was confirmed by double scope to be 1–2 cm distal to the functional narrowing, antegrade measurement based on the SCJ inherently overestimates the apparent gastric myotomy length. This reflects measurement geometry rather than excessive cutting. However, the preservation of the sling fibers during POEM may reduce the GERD risk [18], and anterior myotomy may be preferable where feasible. As sling fibers are involved in the antireflux mechanism, their preservation may help reduce the risk of postoperative GERD. POEM combined with fundoplication is another promising option that could be considered in selected patients, although it is technically more demanding [19]. Given these considerations, a function‐oriented approach, in which the level of functional narrowing is precisely identified using the double‐scope technique and the myotomy is limited to the minimum length required to release this obstruction, may represent the most practical strategy to balance therapeutic efficacy and reflux prevention in this high‐risk phenotype. Although all patients with conclusive GERD were managed conservatively with PPIs or PCABs, this high rate of reflux raises concerns about the long‐term outcomes, such as the development of Barrett's esophagus or adenocarcinoma. Continued endoscopic surveillance and long‐term acid suppression may therefore be warranted in these patients.

From the perspective of the phase concept of the EGJ, originally developed to explain the dynamic antireflux mechanisms, these cases can be interpreted as having relaxation failure primarily at Phase I, which corresponds to the gastric‐side structures such as the clasp fibers [10]. This concept was initially proposed to provide a more comprehensive understanding of the EGJ as a functional reflux barrier beyond the traditional focus on the LES. Although classic achalasia usually involves a dysfunction at Phase II (LES), leading to contractile failure, our findings suggest that the impaired relaxation extends into Phase I in this phenotype. This anatomical involvement of the gastric side may help explain the unusually high incidence of GERD observed in these cases. Consequently, these cases might be better conceptualized as a Phase I‐predominant achalasia.

The present study was limited owing to its small sample size and retrospective design. Notably, all patients in this cohort were male and had straight‐type achalasia; however, it remains unclear whether this reflects a true biological predisposition or sampling bias, and whether the long‐term outcomes, including the risk of Barrett's esophagus, are representative of this phenotype. Moreover, it did not compare the study cases with typical achalasia. Furthermore, all observations were made while the patients were sedated; conscious deep inhalation may increase the SCJ visibility, suggesting that the prevalence of this phenotype might have been underestimated. GERD was assessed endoscopically without routine pH monitoring. However, according to the Lyon consensus [16], Grade ≥ C esophagitis is considered GERD. Therefore, the absence of pH monitoring in the present study is unlikely to have substantially impacted the main study findings. In addition, objective physiologic assessment of EGJ distensibility, such as EndoFLIP, was not available in this retrospective cohort, which limits direct functional validation of distal EGJ relaxation failure. The diagnosis of achalasia was based on the Chicago Classification version 3.0 in combination with esophagram and endoscopic findings; although a small number of cases might be classified as EGJ outflow obstruction under version 4.0, this is unlikely to have influenced the main conclusions.

In conclusion, achalasia with visible SCJ and distal relaxation failure is a rare but distinct entity. This phenotype is associated with an exceptionally high incidence of conclusive post‐POEM GERD following POEM. Despite this, most patients responded to conservative treatment. Recognizing this endoscopic feature may aid in risk stratification and procedural planning, including function‐oriented myotomy planning and, when necessary, adjunctive antireflux measures.

## Author Contributions

Conception and design: Kazuya Sumi and Haruhiro Inoue. Data collection and treatment of patients: Kazuya Sumi, Yohei Nishikawa, Kazuki Yamamoto, Ippei Tanaka, and Manabu Onimaru. Data analysis and interpretation: Kazuya Sumi. Drafting of the article: Kazuya Sumi and Haruhiro Inoue. Critical revision of the article for important intellectual content: Kazuya Sumi, Haruhiro Inoue, Yohei Nishikawa, Kazuki Yamamoto, Ippei Tanaka, Mayo Tanabe, Manabu Onimaru, Koji Otsuka, Takayoshi Ito, and Noboru Yokoyama. Supervision: Haruhiro Inoue. All authors have approved the final draft submitted.

## Funding

The authors have nothing to report.

## Ethics Statement

This retrospective study was approved by the Institutional Review Board of Showa University Koto‐Toyosu Hospital (approval number: 2024‐130‐B) and conducted in accordance with the Declaration of Helsinki.

## Consent

The requirement for informed consent was waived due to the retrospective design.

## Conflicts of Interest

Author H.I. is an advisor of Olympus Corporation and Top Corporation, and has received educational grants from Olympus Corporation and Takeda Pharmaceutical Co. The other authors declare no conflicts of interest related to this article.

## Supporting information


**Video S1:** Endoscopy showing a rare achalasia phenotype with clearly visible circumferential SCJ and narrowing at the gastric folds, where impaired relaxation causes resistance during scope advancement.

## Data Availability

The datasets generated and/or analyzed during the current study are not publicly available due to patient privacy restrictions but are available from the corresponding author upon reasonable request.

## References

[1] H. Inoue , H. Minami , Y. Kobayashi , et al., “Peroral Endoscopic Myotomy (POEM) for Esophageal Achalasia,” Endoscopy 42, no. 4 (2010): 265–271, 10.1055/s-0029-1244080. 20354937

[2] T. Tatsuta , H. Inoue , Y. Shimamura , et al., “Peroral Endoscopic Myotomy in Spastic Esophageal Disorders: Clinical Outcomes and Optimal Approaches,” Digestive Endoscopy 37 (2025): 758–765, 10.1111/den.15008. 40094186 PMC12244300

[3] K. Hugova , J. Mares , B. Hakanson , et al., “Endoscopic or Surgical Myotomy in Patients With Idiopathic Achalasia,” New England Journal of Medicine 381, no. 23 (2019): 2219–2229, 10.1056/nejmoa1905380. 31800987

[4] H. Shiwaku , H. Sato , Y. Shimamura , et al., “Risk Factors and Long‐Term Course of Gastroesophageal Reflux Disease After Peroral Endoscopic Myotomy: A Large‐Scale Multicenter Cohort Study in Japan,” Endoscopy 54, no. 9 (2022): 839–847, 10.1055/a-1753-9801. 35172368

[5] V. Kumbhari , P. Familiari , N. Bjerregaard , et al., “Gastroesophageal Reflux After Peroral Endoscopic Myotomy: A Multicenter Case–Control Study,” Endoscopy 49, no. 7 (2017): 634–642, 10.1055/s-0043-105485. 28472834

[6] E. M. Wessels , G. M. Masclee , B. A. Bastiaansen , P. Fockens , and A. J. Bredenoord , “Incidence and Risk Factors of Reflux Esophagitis After Peroral Endoscopic Myotomy,” Neurogastroenterology and Motility 36, no. 6 (2024): e14794, 10.1111/nmo.14794. 38587128

[7] K. Iwakiri , Y. Hoshihara , N. Kawami , et al., “The Appearance of Rosette‐Like Esophageal Folds (“Esophageal Rosette”) in the Lower Esophagus After a Deep Inspiration Is a Characteristic Endoscopic Finding of Primary Achalasia,” Journal of Gastroenterology 45, no. 4 (2010): 422–425, 10.1007/s00535-009-0179-7. 20013295

[8] K. Gomi , H. Inoue , H. Ikeda , et al., “New Endoscopic Classification of the Cardiac Orifice in Esophageal Achalasia: Champagne Glass Sign,” Digestive Endoscopy 28, no. 6 (2016): 645–649, 10.1111/den.12642. 26969481

[9] K. Sumi and H. Inoue , “Gastrointestinal: Esophageal Achalasia With Unusual Endoscopic Findings,” Journal of Gastroenterology and Hepatology 40, no. 3 (2025): 559–561, 10.1111/jgh.16852. 39694042

[10] K. Sumi and H. Inoue , “Achalasia Characterized by Gastric Folds and Squamocolumnar Junction,” Internal Medicine 65 (2025): 621–622, 10.2169/internalmedicine.5893-25. 40707228 PMC12979632

[11] H. Inoue , M. Tanabe , Y. Shimamura , et al., “Phase Concept: Novel Dynamic Endoscopic Assessment of Intramural Antireflux Mechanisms (With Video),” Digestive Endoscopy 37, no. 3 (2025): 257–265, 10.1111/den.14922. 39307960 PMC11884963

[12] Japan Esophageal Society , “Descriptive Rules for Achalasia of the Esophagus, June 2012,” Esophagus 14, no. 4 (2017): 275–289, 10.1007/s10388-017-0589-1. 28983228 PMC5603650

[13] V. F. Eckardt , “Clinical Presentation and Complications of Achalasia,” Gastrointestinal Endoscopy Clinics of North America 11, no. 2 (2001): 281–292, 10.1016/s1052-5157(18)30071-0. 11319062

[14] P. J. Kahrilas , A. J. Bredenoord , M. Fox , et al., “The Chicago Classification of Esophageal Motility Disorders, v3.0,” Neurogastroenterology and Motility 27, no. 2 (2015): 160–174, 10.1111/nmo.12477. 25469569 PMC4308501

[15] L. R. Lundell , J. Dent , J. R. Bennett , et al., “Endoscopic Assessment of Oesophagitis: Clinical and Functional Correlates and Further Validation of the Los Angeles Classification,” Gut 45, no. 2 (1999): 172–180, 10.1136/gut.45.2.172. 10403727 PMC1727604

[16] C. P. Gyawali , R. Yadlapati , R. Fass , et al., “Updates to the Modern Diagnosis of GERD: Lyon Consensus 2.0,” Gut 73, no. 2 (2024): 361–371, 10.1136/gutjnl-2023-330616. 37734911 PMC10846564

[17] J. Lyons , C. Boutros , S.‐Z. Khan , J. Benson , D. A. Hashimoto , and J. Marks , “Preoperative Patient Factors and Anatomy Do Not Predict Who Will Develop Reflux After Per Oral Endoscopic Myotomy,” Surgical Endoscopy 37, no. 9 (2023): 7178–7182, 10.1007/s00464-023-10205-8. 37344752

[18] H. Shiwaku , H. Inoue , A. Shiwaku , H. Okada , and S. Hasegawa , “Safety and Effectiveness of Sling Fiber Preservation POEM to Reduce Severe Post‐Procedural Erosive Esophagitis,” Surgical Endoscopy 36, no. 6 (2022): 4255–4264, 10.1007/s00464-021-08763-w. 34716481

[19] H. Inoue , A. Ueno , Y. Shimamura , et al., “Peroral Endoscopic Myotomy and Fundoplication: A Novel NOTES Procedure,” Endoscopy 51, no. 2 (2019): 161–164, 10.1055/a-0820-2731. 30654395

