# Supplementary material for: An Exploratory Endoscopic Achalasia Phenotype With Visible Squamocolumnar Junction and Distal Esophagogastric Junction Relaxation Failure: Association With Gastroesophageal Reflux Disease After Peroral Endoscopic Myotomy (With Video)
Source: Dig Endosc. 2026 Apr 4;38(4):e70149. doi: 10.1111/den.70149 (PMC13049693; doi:10.1111/den.70149)
Supplement: Supplementary file 1 — Video S1: Endoscopy showing a rare achalasia phenotype with clearly visible circumferential SCJ and narrowing at the gastric folds, where impaired relaxation causes resistance during scope advancement. [file DEN-38-0-s001.zip › den70149-sup-0002-Supinfo2@video text.docx]

**Video text.**

A small amount of esophageal residue was noted. The esophagus was dilated, with typical endoscopic findings of achalasia. As the scope advanced distally, the squamocolumnar junction became clearly visible, and a functional narrowing was noted just beyond it, with visible gastric folds on the distal side. Resistance was encountered during passage through this area, and the upper border of the gastric folds was clearly identified.
